# An Improved Step Detection Algorithm for Indoor Navigation Problems with Pre-Determined Types of Activity

**DOI:** 10.3390/s25206358

**Published:** 2025-10-14

**Authors:** Michał Zieliński, Andrzej Chybicki, Aleksandra Borsuk

**Affiliations:** 1 Faculty of Electronics, Telecommunications and Informatics, Gdańsk University of Technology, 80-233 Gdańsk, Poland; s184372@student.pg.edu.pl (M.Z.); s184620@student.pg.edu.pl (A.B.); 2 Department of Geoinformatics, Gdańsk University of Technology, 80-233 Gdańsk, Poland

**Keywords:** indoor navigation, step detection, LSTM, sensor fusion, mobile application

## Abstract

Indoor navigation (IN) systems are increasingly essential in environments where GPS signals are unreliable, such as hospitals, airports, and large public buildings. This study explores a smartphone-based approach to indoor positioning that leverages inertial sensor data for accurate step detection and counting, which are fundamental components of pedestrian dead reckoning. A long short-term memory (LSTM) network was trained to recognize step patterns across a variety of indoor movement scenarios. The generalized model achieved an average step detection accuracy of 93%, while scenario-specific models tailored to particular movement types such as turning, stair use, or interrupted walking achieved up to 96% accuracy. The results demonstrate that incorporating activity-specific training improves performance, particularly under complex motion conditions. Challenges such as false positives from abrupt stops and non-walking activities were reduced through model specialization. Although the system performed well offline, real-time deployment on mobile devices requires further optimization to address latency constraints. The proposed approach contributes to the development of accessible and cost-effective indoor navigation systems using widely available smartphone hardware and offers a foundation for future improvements in real-time pedestrian tracking and localization.

## 1. Introduction

Indoor navigation (IN) refers to the use of technology to determine and track the position of people or objects within buildings, in places where traditional GPS signals are unreliable or unavailable such as airports, commercial buildings, warehouses, and public estates. Due to its wide range of potential applications, including emergency response, smart buildings, healthcare, and location-based services in large facilities, indoor navigation is a promising and dynamic field of research [1]. Existing IN solutions often suffer from high costs or low accuracy. For instance, approaches using beacon-based systems require dedicated hardware installation and software integration, imposing significant financial and logistical burdens on building owners. Conversely, GPS technology is inherently imprecise for indoor environments due to signal attenuation and multipath effects. A widely accessible smartphone-based indoor navigation application may be able to enhance the efficiency of public institutions, such as hospitals and municipal buildings, by providing a cost-effective alternative for wayfinding solutions. Many approaches have been considered, including wearable sensors [1,2,3], computer vision solutions [4], and inertial measuring units (IMUs) [5,6,7,8,9,10]. However, developing such an application fit for real world applications necessitates addressing several challenges, including detecting movement modes (e.g., walking, using stairs, or elevators) and accurately estimating traveled distances.

In this context, the present study focuses on fundamental aspects of smartphone-based odometry: step detection and step counting, which are essential for reliable distance estimation in indoor navigation systems. Combined with motion orientation data derived from the magnetometer, this allows precise determination of the distance traveled by the user from predefined control points within a building.

In this paper, we propose a novel approach to indoor positioning, which is complemented by information about the user’s activity, potentially obtained using established activity recognition methods as explored in prior works [11,12,13,14,15,16,17]. This enhancement is intended to improve step-counting accuracy using smartphone sensors, thereby contributing to the development of robust and cost-effective indoor positioning solutions.

This paper is organized into five main sections. The Introduction outlines the motivation for smartphone-based indoor navigation and the challenges of step detection. The Section 2 presents a review of existing techniques and describes the proposed LSTM-based approach, data collection setup, and test scenarios. The Section 3 reports the performance of generalized and specialized LSTM models across different movement types. The discussion provides a detailed analysis of model strengths and weaknesses, including error sources and efficiency considerations. Finally, the conclusions summarize the contributions, highlight practical limitations, and suggest directions for future work.

## 2. Materials and Methods

### 2.1. State of the Art Applications of Machine Learning in Indoor Navigation

Indoor navigation has seen significant advances in recent years, driven largely by the rapid development of machine learning techniques. In particular, wireless signal sensing technologies combined with machine learning have been leveraged to infer location and occupancy without relying on GPS. Polák et al. [18] introduced a fingerprinting approach based on Bluetooth low energy (BLE) received signal strength indicators (RSSI) from multiple anchors and radio channels, demonstrating that random forest classifiers can achieve over 99% accuracy in both one- and two-dimensional environments. More recently, Želený et al. [19] explored room occupancy detection using passive Wi-Fi probe requests and evaluated several neural network architectures (CNN, LSTM, GRU), finding that a GRU-based model achieved a 91.8% accuracy for presence detection and 75.1% for estimating occupancy levels. Beyond radio frequency (RF)-based fingerprinting approaches, recent research has also explored alternative sensing modalities and forward-looking communication technologies for indoor localization. For instance, a 2025 study introduced an efficient deep learning-based device-free localization framework using passive infrared (PIR) sensors [20]. By combining CNN-LSTM models with ensemble learning (bagging), the system achieved a mean localization error of approximately 0.55 m, with 80% of errors below 0.8 m; that work highlights the potential of non-RF, low-cost sensors for multi-person localization in device-free scenarios. In parallel, advances in 6G communication technologies are opening new opportunities for highly accurate positioning. A 2024 study proposed a deep learning framework leveraging channel state information (CSI) at terahertz frequencies, enriched with attention mechanisms such as self-attention and channel attention [21]. Their results demonstrate the ability of attention-enhanced CNN models to capture fine-grained signal structures, underscoring the promise of next-generation communication infrastructure for achieving sub-meter indoor localization accuracy.

### 2.2. Step-Detection—Overview of Techniques and Knowledge

The challenge of detecting and counting steps has been widely studied, leading to a range of methods from simpler peak detection algorithms with pre- and post-processing enhancements to advanced machine learning-based approaches. Below, a brief review of selected open-source solutions and relevant literature is presented.

Most common approaches in the early stages of development of step-counting methods relied on peak detection within specific time windows. These methods are relatively simple to implement and use filtering techniques with predefined parameters based on testing results [22,23]. For example, Mladenov et al. [24] utilized combined three-axis accelerometer data to derive a magnitude, which was then filtered using a low-pass filter and processed within time buffers to detect steps. A more complex five-step pipeline was proposed by D. Salvi et al. [25], consisting of pre-processing, filtering, scoring, detection, and post-processing. The techniques used in these stages require parameters determined through trial and error, making the method somewhat inconsistent in real-life applications. This method was tested with various phone placements including the front and back pocket, in hand, in an armband, a purse, and a neck pouch, revealing that the optimal parameters varied for each position.

Both of the aforementioned methods are prone to falsely detecting steps when the user engages in activities that mimic walking, such as playing games or texting. To address this issue, an approach was proposed [26] that introduces constraints based on step features: periodicity, similarity, and continuity. This method aims to reduce noise caused by false walking. The process begins with detecting a peak and identifying the motion state within a given time window. The detected step is then compared to the previous one in terms of periodicity, which considers the time difference between successive steps, and similarity, which measures how closely two acceleration peaks match, with greater similarity corresponding to smaller differences in their values. The continuity constraint is based on the assumption that motion states, such as walking, running, or standing, generally remain smooth and uninterrupted for a certain duration, whereas false walking often produces irregular, non-continuous motion data. Finally, the number of steps is estimated by counting acceleration peaks that satisfy all three constraints.

Numerous studies [27,28,29,30,31,32] have investigated step detection using neural networks; notably, a recent study [33] focused on step detection using neural network models applied to accelerometer and gyroscope data, investigating two distinct approaches. The first approach utilized convolutional neural networks (CNNs) to extract spatial features from time-series data, enabling precise localization of step events. This method demonstrated higher accuracy and improved inference speed compared to previously used long short-term memory (LSTM) models, suggesting that CNNs are more effective in capturing spatial dependencies in step detection tasks. The second approach adopted a YOLO-like architecture designed to predict steps in start–end pairs, which reduced the need for post-processing and enhanced real-time processing efficiency. The CNN-based models outperformed the LSTM models in terms of accuracy, while the YOLO-like models were more suitable for real-time applications due to their faster inference times.

Another project [34] implemented a full pipeline for step detection using a random forest classifier on labeled gait data. The model achieved over 90% accuracy, precision, recall, and F1-score by selecting important features. It was trained using the OU-ISIR biometric dataset [35] containing labeled gait data from over 460 people. The pipeline includes data preprocessing, feature extraction using recursive feature elimination, model training, and evaluation.

Recent state-of-the-art research in smartphone-based step detection has demonstrated substantial improvements in robustness and accuracy through the integration of advanced signal processing and machine learning methods. For instance, a CEEMDAN-HT–based approach combined with support vector machines (SVM) achieved 98.10% accuracy in free walking and 93.85% in false walking scenarios by adaptively modeling mixed gaits and smartphone postures while reducing noise through empirical mode decomposition [36]. Similarly, the MCCIF-SDE method employed adaptive peak-valley detection with dynamic window constraints, enhanced by gait similarity measures, reaching 96.5% accuracy and maintaining a low 1.8% step length estimation error across varying user conditions, without relying on complex machine learning parameter tuning [37]. Another study used data collected using an iPhone and proposed an adaptive step detection method based on a time-dependent decay mechanism has shown 97.4% accuracy under diverse smartphone placements and pedestrian patterns, while also meeting low-latency requirements critical for real-time indoor positioning [38]. Together, these methods highlight a trend toward hybrid frameworks that leverage both traditional signal processing and machine learning to achieve robust step detection.

Building on these advances, recent studies have further emphasized the importance of evaluating step detection performance under diverse pedestrian activities, including not only flat walking but also stairs, slopes, and turning movements. Li et al. [39] proposed a smartphone-based algorithm that combines a variable sliding window with an adaptive peak threshold to address the variability of unconstrained device carrying, achieving step-counting accuracies above 98% across different types of devices (21 smartphone models). Their evaluation included flat walking, stair ascent, and stair descent, demonstrating strong robustness across different movement contexts. Tiwari and Jain [40] introduced a Dynamic Weight Integrated Fuzzy C-Means (DWIFCM) clustering approach that leveraged accelerometer-derived statistical features to distinguish step patterns from noise, and validated it on a wide range of activities including flat walking, slopes, turns, and stair walking, showing improved robustness across varying terrains compared to baseline methods. Extending beyond step detection to activity context, Shaikh et al. [41] developed an embedded inertial-sensor system using IMUs placed near the knee, which jointly classifies terrain (flat walking, stair ascent, stair descent) and detects gait events in real time, achieving classification accuracies near 99% while operating efficiently on a microcontroller platform. Collectively, these studies demonstrate the growing ability of step detection methods to operate reliably under diverse activity scenarios, paving the way for broader real-world applications.

### 2.3. Proposed Approach

In this context the objective of research presented in the paper was to develop an improved step detection mechanism for indoor navigation, explicitly accounting for the user’s behavior and the type of activity performed. The intention was to design and evaluate methods using a set of long short-term memory networks that could be widely applicable in practice, without the need to develop or integrate additional sensors or technologies, as has frequently been the case in previous studies.

The step detection itself was based on the analysis of inertial sensor signals recorded by the smartphone’s built-in accelerometer and gyroscope. Specifically, we used the accelerometer magnitude (calculated as the Euclidean norm of the three accelerometer axes) as the primary indicator for detecting step events, since this reflected the overall intensity of motion independent of phone orientation.

To achieve this goal, we first created an environment for collecting research data and annotating it for subsequent neural network training. This chapter describes these aspects in detail.

The test environment consists of two main components (both of which have been released as open-source), as follows:

- A mobile application that records sensor data and activity parameters (steps, type of activity);
- A server application that stores collected data, which was later processed and used for training.

In the next step of this research, we conducted a series of controlled tests designed to capture various walking patterns and user behaviors. The data was collected independently by two different users using two devices (Samsung Galaxy M23 5G and Samsung Galaxy A45 5G by: Samsung Electronics, Suwon, Republic of Korea). The tests were structured to simulate real-world scenarios, ensuring the collected data reflects the complexity of indoor navigation. The collected time-series data served as input for the LSTM model, allowing it to learn complex step patterns and user activity dependencies. The tests were split into train and test sets in an 80:20 ratio.

We performed a total of 30–40 repetitions of each test type to ensure statistical significance and consistency. The tests were conducted by two participants using two different smartphone models to account for hardware variability. During the test, the smartphone was held in the participant’s hand at approximately chest height and slightly in front of the body, mimicking typical usage while following navigation instructions. The sensor sampling rate was maintained at approximately 200 Hz to ensure high-resolution data collection. The following test types were performed:

1. Flat 10: 10 steps in a straight line. A baseline test to evaluate the accuracy of straightforward step counting;
2. Interrupted 3 × 3: 3 steps, pause, 3 steps, pause, 3 steps. Designed to test the algorithm’s ability to handle interrupted walking patterns;
3. Left Turn: 5 steps, left turn, 5 steps. Introduces a change in direction to assess the sensitivity of the step detection mechanism to turns;
4. Right Turn: 5 steps, right turn, 5 steps. Similar to the previous test but involving a right turn to evaluate symmetry in directional changes;
5. Stairs Transition: 5 steps up/down stairs, 3 flat steps, 5 steps. Designed to simulate walking up and down stairs with a transition to flat ground, testing the mechanism’s ability to adapt to elevation changes;
6. Stairs: 5 steps up/down stairs. A simplified version of the previous test focused solely on elevation changes.

The selection of test types was not driven by a strict theoretical framework but by a practical motivation to reflect typical movement patterns relevant to indoor navigation. Straight walking, interrupted walking, turns, and stair transitions represent common real-world scenarios that significantly affect inertial sensor signals, providing a controlled yet representative basis for evaluating step-detection performance.

**Figure 1:**
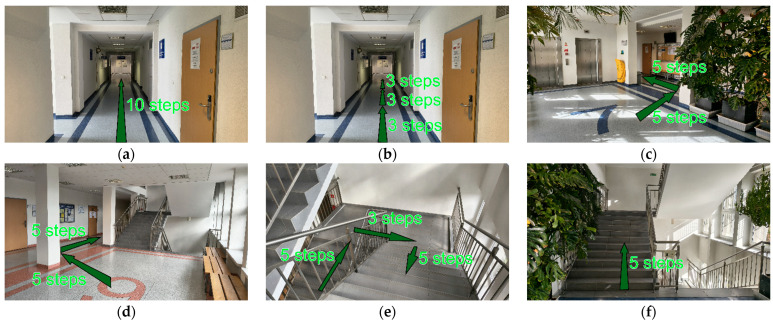
The test environment for each of test types: (**a**) Flat 10; (**b**) Interrupted 3 × 3; (**c**) Left Turn; (**d**) Right Turn; (**e**) Stairs Transition; (**f**) Stairs.

The tests were conducted at the Gdańsk University of Technology, in the Faculty of Electronics, Telecommunications and Informatics building (Figure 1). At the time of data collection, the space was mostly empty, which minimized disturbances and ensured more predictable results. The surface of the corridor was vinyl and the stairs were covered in stone tiles.

**Figure 2:**
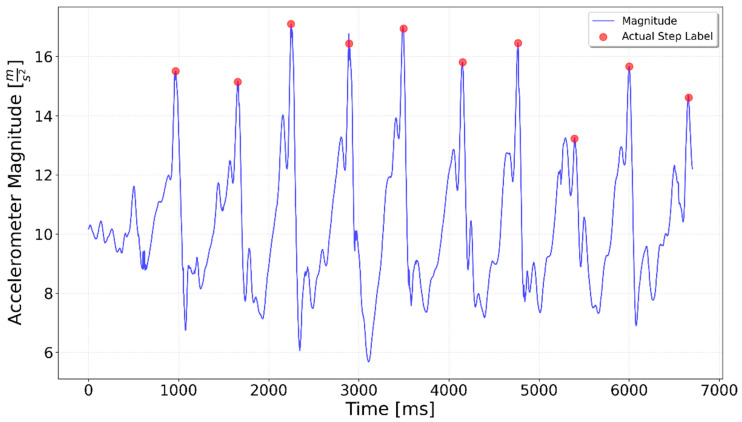
Results of step detection for ‘10 steps’ test. Step detection moments are indicated by red dots on the chart. The blue line represents the accelerometer magnitude value. A sequence of 10 evenly spaced steps can be clearly observed.

**Figure 3:**
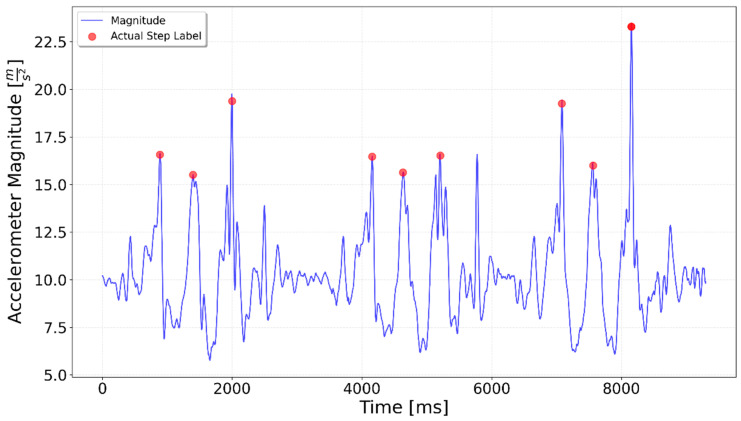
Interrupted 3 × 3. The three series of steps are separated by approximately 1.5 s of idle time each, which is visible as lower accelerometer magnitude values.

**Figure 4:**
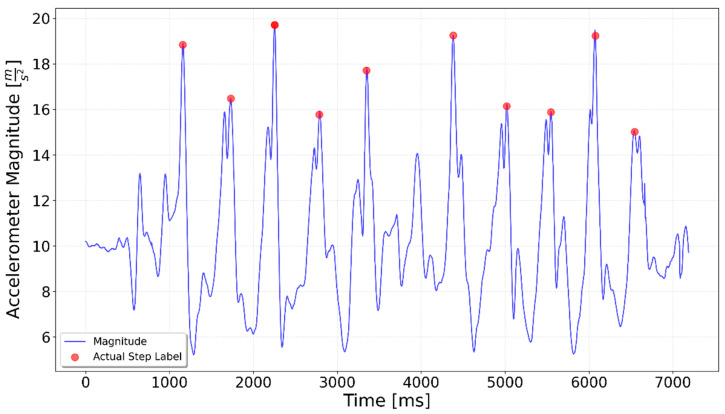
A combined test including 5 steps, left turn, and 5 steps. The left turn happens around the 4000 ms time mark and is indicated by a spike in the magnitude value.

**Figure 5:**
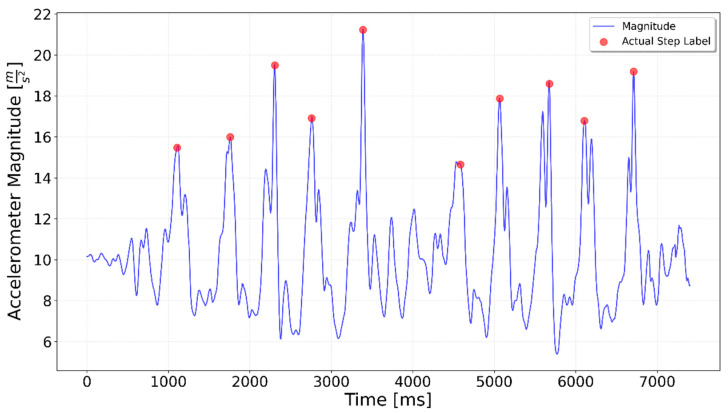
A combined test including 5 steps, right turn, 5 steps. The right turn occurs around the 4000 ms mark but does not produce a spike in the magnitude value.

**Figure 6:**
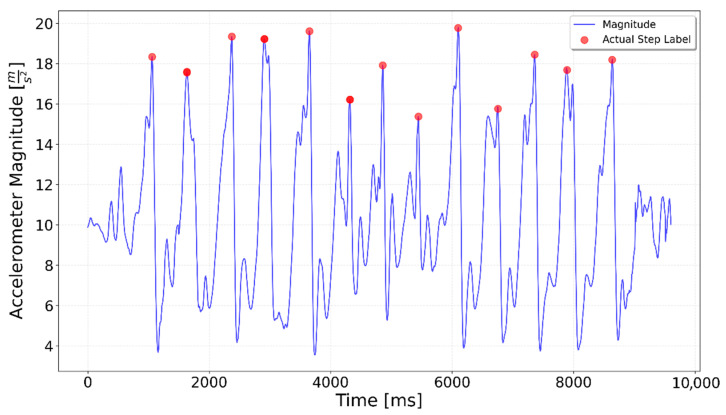
Test including 5 steps up the stairs, 3 on flat, 5 steps up again. The 3 flat-surface steps occur between the 4000 ms and 6000 ms marks, preceded and followed by the stair-climbing segments.

**Figure 7:**
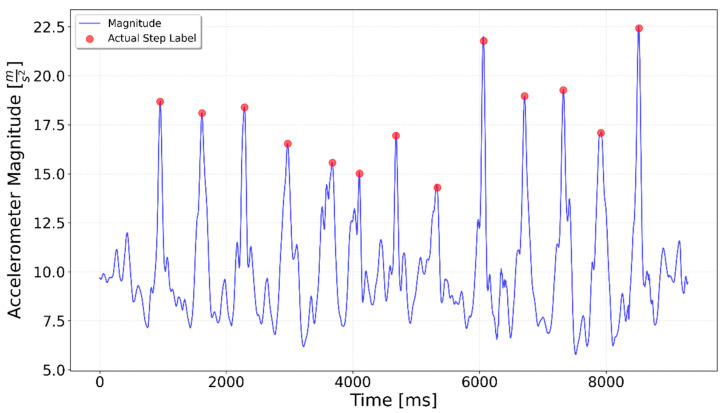
Test including 5 steps down the stairs, 3 on flat, 5 steps down again. The 3 flat-surface steps occur between the 4000 ms and 5800 ms marks, preceded and followed by stair-descending segments.

**Figure 8:**
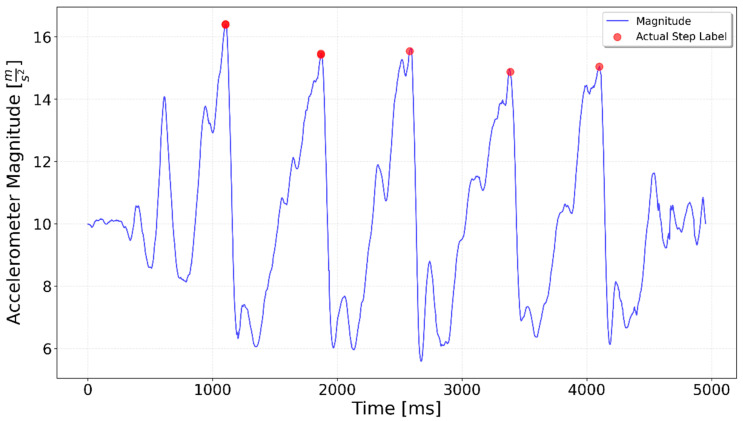
Test including 5 steps up stairs. Five distinct peaks, corresponding to five upward steps, are visible, but the peak magnitudes are generally lower than those seen during ascent.

**Figure 9:**
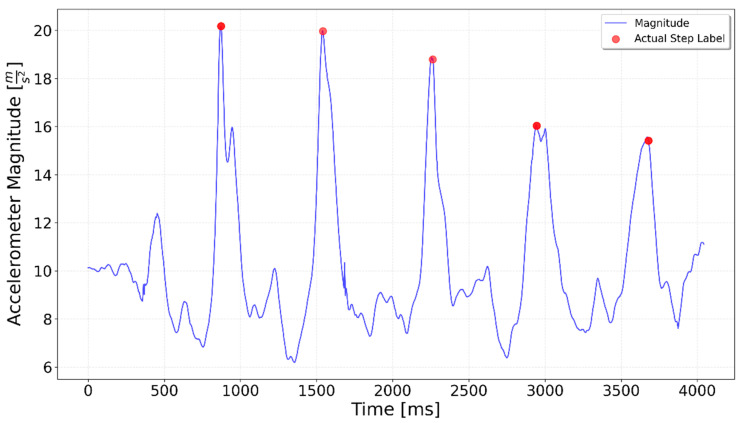
Test including 5 steps downstairs. Five distinct peaks are visible for the downward steps, with higher magnitude values reflecting the increased impact of stair descent.

Figure 2, Figure 3, Figure 4, Figure 5, Figure 6, Figure 7, Figure 8 and Figure 9 display the results for each test type. In each graph, the *x*-axis represents the time elapsed during the test, while the *y*-axis shows the accelerometer magnitude, a key parameter for step detection. The red dots indicate manually labeled steps, which were annotated during the data labeling process.

The LSTM network was implemented using PyTorch v2.5.1. The model consists of two LSTM layers with a hidden size of 64 and a dropout rate of 0.2 to reduce overfitting. The output of the last LSTM time step is passed through a fully connected (linear) layer to produce a single output value, representing the step prediction. A binary classification approach was used, where the output indicated whether a step was present or not.

**Figure 10:**
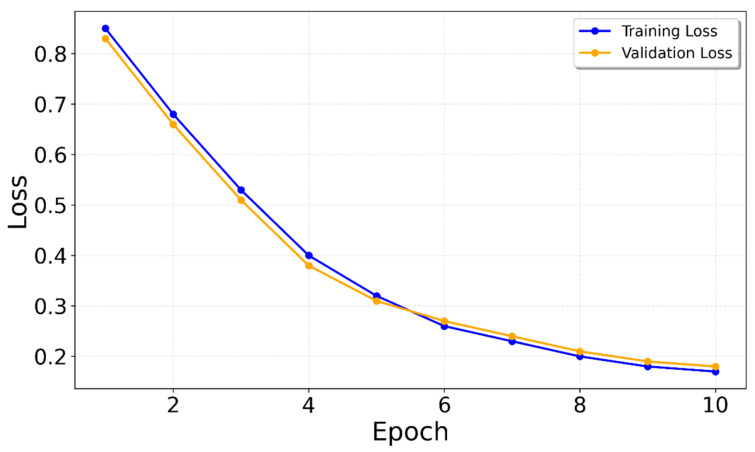
Validation and training loss for the LSTM.

**Figure 11:**
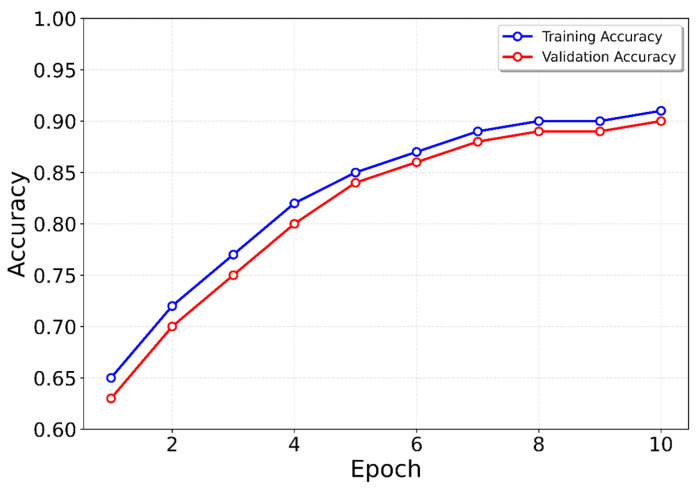
Validation and training accuracy for the LSTM.

The model was trained using the Adam optimizer with a learning rate of 0.001 (see Figure 10 and Figure 11). To handle the class imbalance between step and non-step samples, a weighted binary cross-entropy loss (BCEWithLogitsLoss) was applied, with the positive class (steps) weighted inversely proportional to its occurrence in the training set. This helped balance the contribution of step and non-step samples during training.

During evaluation, the model’s output was thresholded at 0.5 to determine step predictions. To account for variability in real-world step timing, a leeway mechanism was introduced. The sampling rate was set to 100 Hz, and a time leeway of 100 milliseconds was allowed when matching predicted steps to actual steps. A prediction was considered correct if it matched the label within this window.

The total number of real steps and counted steps was also tracked to assess the model’s overall performance. By introducing leeway and handling class imbalance, the model was designed to provide robust step detection under varying walking conditions and across different hardware platforms.

## 3. Results

The test split of the dataset was used to verify the quality of the LSTM network in the step detection task.

Table 1 presents the results of step detection across different walking scenarios for a generalized LSTM. The first column, “Type” lists the walking scenario under evaluation. The second column, “Real per type” shows the total number of actual steps recorded across all repetitions of each scenario. The third column, “Detected per type” contains the number of steps identified by the detection algorithm. The fourth column, “Accuracy” is the success rate of step detection, calculated as the proportion of correctly detected steps. The fifth column, “Recall” represents the completeness of positive predictions, calculated as the proportion of actual steps that were successfully detected by the system. The sixth column, “F1-Score” is the harmonic mean of precision and recall. A step was considered correctly detected if its predicted timestamp fell within a 100-millisecond leeway from the corresponding actual step, accounting for natural variability in step timing.

Table 2 presents another approach; each respective type used a specialized LSTM trained only on the given scenario.

## 4. Discussion

The average accuracy for the generalized LSTM was 93%, with the lowest observed for the Interrupted 3 × 3 test, where more steps than expected were detected. The highest accuracy was achieved for the Left Turn and Right Turn tests, suggesting that turning in place did not negatively affect step detection.

The accuracy of scenario-specific models is significantly higher, averaging 96%, with notable improvements observed in the Interrupted 3 × 3, Transition, and Flat 10 scenarios. These results suggest that tailoring LSTM models to specific walking patterns can yield substantial performance gains in step detection, particularly in complex or transitional movement conditions.

### 4.1. Generalized Network Approach

**Figure 12:**
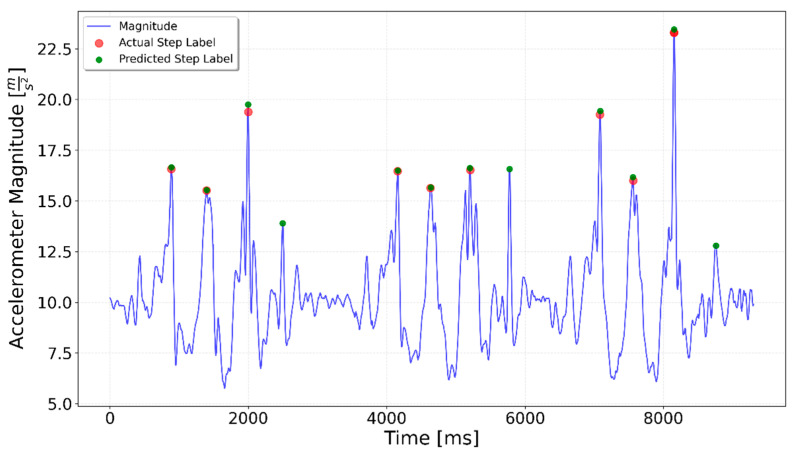
Prediction for a 3 × 3 test. Green dots indicate the generalized LSTM-predicted steps, red dots show ground truth steps, and the blue line represents accelerometer magnitude. Ideally, green and red dots overlap. Each 3-step sequence includes one additional predicted step at the end.

In one of the Interrupted 3 × 3 tests (see Figure 12) the model consistently detected four steps in each segment instead of the expected three. This discrepancy is probably due to the abrupt nature of the stopping motion. When the participant stopped suddenly, the sharp deceleration may have generated sensor signals resembling the characteristics of a step, leading the model to misclassify the stopping motion as an additional step. This suggests that the model is sensitive not only to the regular step pattern but also to transient accelerations and decelerations.

**Figure 13:**
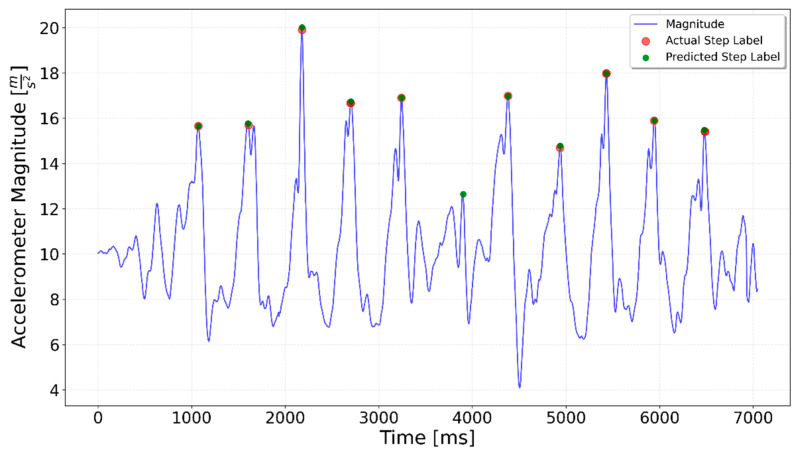
Left Turn test prediction. A small peak that occurred during the turn between the 5-step series was incorrectly interpreted as a step by the model.

**Figure 14:**
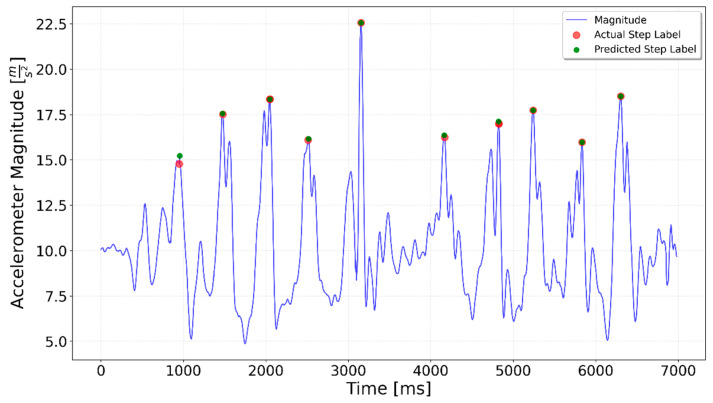
Right Turn test prediction. In some turning trials, the peak during the turn was absent, resulting in a more accurate step prediction.

In one of the Left Turn tests (Figure 13), the model incorrectly detected a step at the midpoint of the turn. This misclassification occurred probably because the sensor signals generated during the turning motion resembled the acceleration patterns of a step. Figure 14 illustrates an example without a peak during the turn.

**Figure 15:**
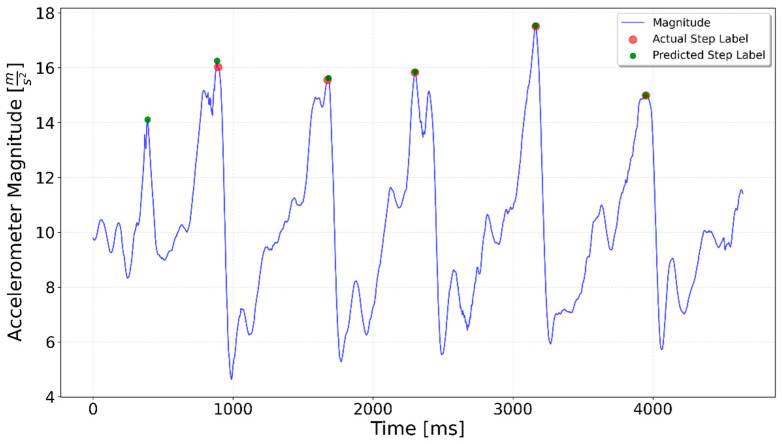
Downstairs test result. The first green dot represents an additional step which was incorrectly detected at the start, the rest correctly overlap with the ground truth.

**Figure 16:**
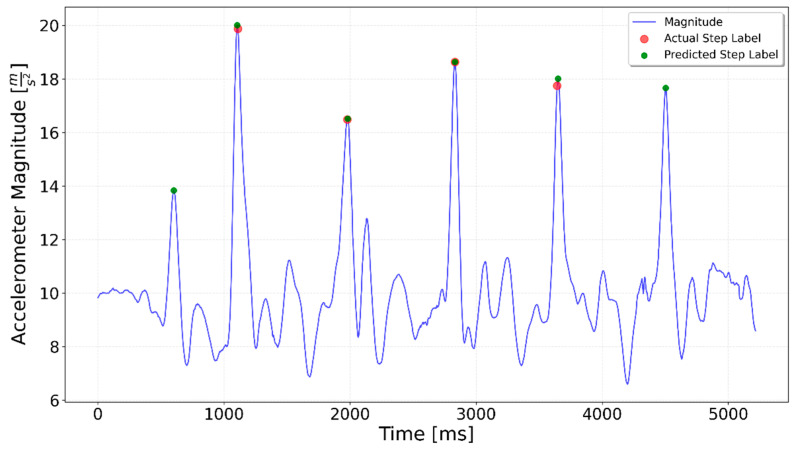
Result for an Upstairs test. A false positive step was identified at the start of the test.

**Figure 17:**
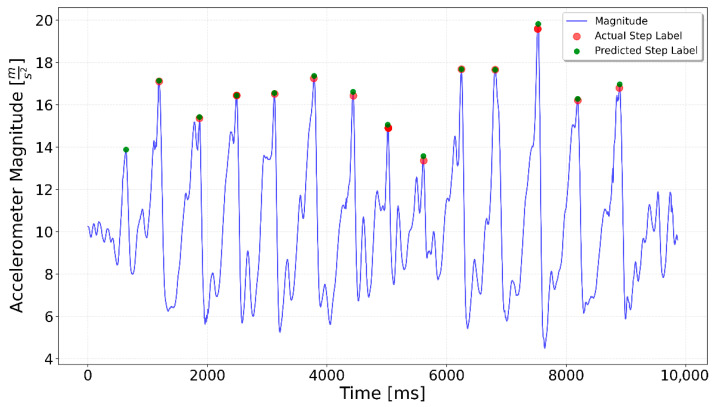
Upstairs Transition test with the generalized network. A step was incorrectly predicted at the start of the test.

In some of the tests, for example, in the downstairs and upstairs tests showcased in Figure 15, Figure 16 and Figure 17, an additional step was detected at the beginning. This is likely to have been caused by a hand movement after selecting the type of test in the mobile app. This caused a spike in the accelerometer and gyroscope magnitude, which could be interpreted as a step.

**Figure 18:**
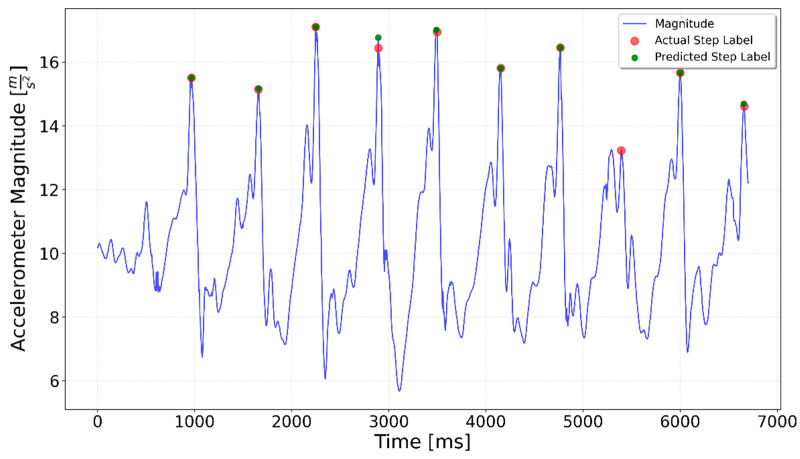
Flat 10 test result. The 8th step was not detected.

The LSTM network failed to recognize the eighth step in the Flat 10 sequence shown in Figure 18, possibly because the accelerometer magnitude was significantly lower than the average for the other steps in the series.

**Figure 19:**
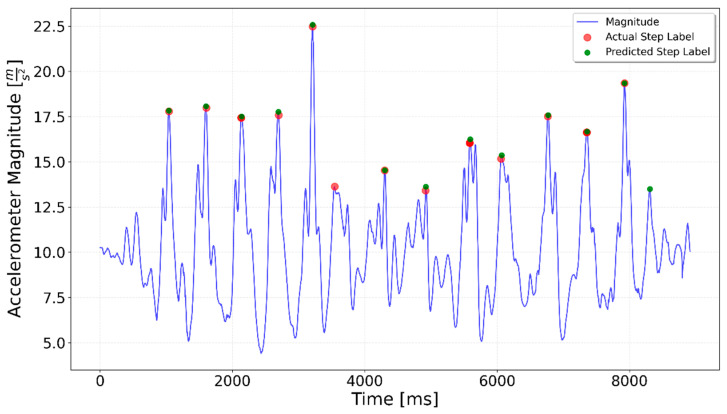
Downstairs Transition step result. The first transition step was not detected, and an additional step was detected while stopping.

An additional step was detected at the end of the Downstairs Transition step in Figure 19. This may have been caused by an abrupt stop at the end, similar to the 3 × 3 Interrupted case shown in Figure 12.

### 4.2. Specialized Network Approach

The specialized LSTM networks achieved higher accuracy compared to the generalized approach. While the generalized model provided solid baseline results, it was prone to false positives and missed steps in irregular scenarios. The specialized models, trained on activity-specific data, reduced these errors by better capturing the unique features of each movement type. This section presents examples of these improvements in detection performance.

**Figure 20:**
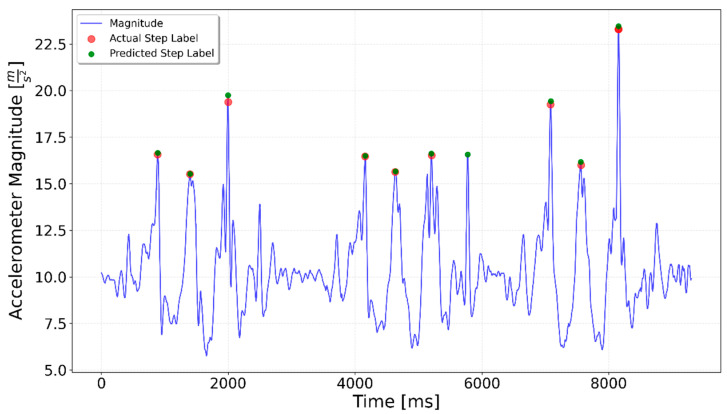
Interrupted 3 × 3 specialized LSTM test result. The fine-tuned model predicted an extra step only in the second series.

In the Interrupted 3 × 3 case, shown in Figure 12, detection improved notably when using a fine-tuned network. In a specialized network approach (see Figure 20) only one additional step was erroneously detected (around time 5750 ms time), whereas all the remaining sequences were handled correctly.

**Figure 21:**
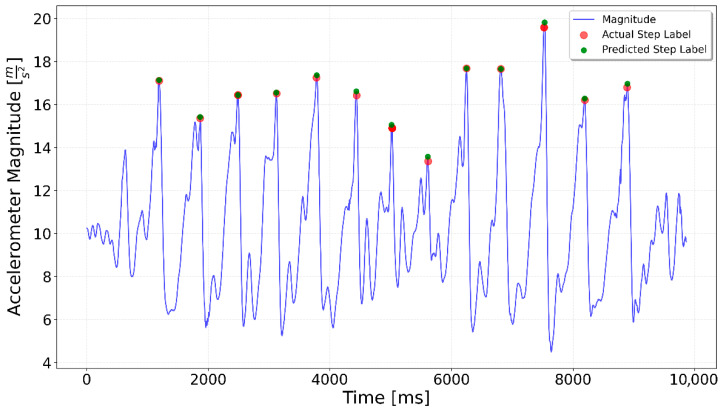
Upstairs Transition specialized LSTM test result. All the steps were correctly identified.

The specialized network was less prone to detecting additional steps at the beginning of the trials. This behavior indicates that the model is more accurate in distinguishing between actual steps and noise during the initial phase. Figure 21 shows the improvements in comparison to the Upstairs Transition test presented in Figure 17. In this case, a false-positive detection at the beginning of the test was eliminated by specialized network.

The new specialized LSTM network also successfully addressed the issue of missed steps, including the previously unrecognized eighth step in the Flat 10 sequence.

**Figure 22:**
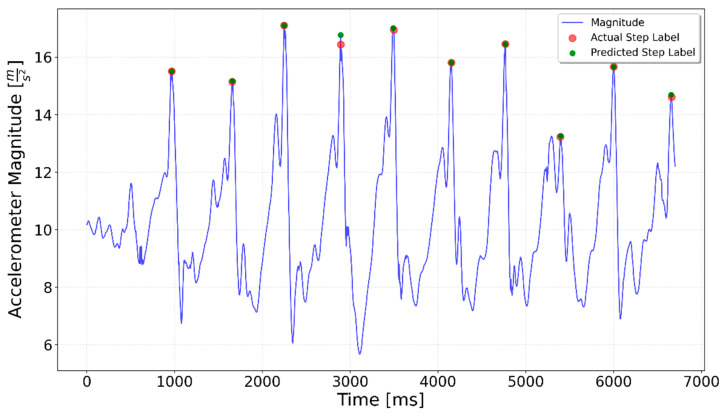
Flat 10 specialized LSTM test result. The step was detected regardless of the magnitude change.

In summary, both the graphical results (Figure 20, Figure 21 and Figure 22) and the quantitative evaluation (Table 1 and Table 2) demonstrated clear improvement when using specialized LSTM networks for step detection in specific activity types. Compared to the generalized model, the specialized networks not only increased overall accuracy but also mitigated typical sources of error such as false positives during irregular movements or missed steps in sequences with low signal magnitude. These findings underline the advantage of tailoring models to distinct motion scenarios, highlighting the potential of activity-specific approaches to enhance the reliability of step detection in real-world indoor navigation applications.

### 4.3. Algorithm Inference Efficiency Considerations

**Figure 23:**
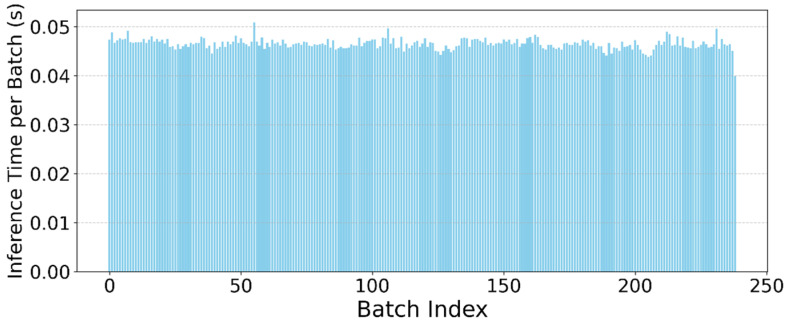
Per-sample inference time (in seconds) over a 160-s sliding window for the LSTM-based step detection model on the Samsung Galaxy A42 5G.

To evaluate the effectiveness of the proposed approach, we conducted an analysis of how the method could be applied in real-world, real-time applications. Accordingly, a performance test of the proposed approach was performed. The LSTM model demonstrated an inference time of approximately 0.5 s for every 1 s of input data when executed on an NVIDIA GeForce RTX 3060 Ti GPU. This indicates that the model operated at half real-time speed under current hardware conditions. While this performance is adequate for offline analysis and prototype-level evaluation, further optimization will be required to enable fully real-time operation, particularly on resource-constrained platforms such as mobile devices. Strategies such as model quantization, pruning, or conversion to more lightweight architectures (e.g., GRU or temporal convolutional networks) may facilitate deployment on edge devices. Additionally, utilizing on-device machine learning acceleration frameworks could further enhance inference efficiency and enable practical real-time step detection in mobile applications. To evaluate this, the model was dynamically quantized, traced using TorchScript, optimized for mobile, and deployed to a Samsung Galaxy A42 5G smartphone. In this mobile CPU-only setting, the quantized model achieved an average inference time of approximately 1.3 s per 1 s of input data, indicating that further compression or architectural simplification may be needed for truly real-time performance on mid-range devices. Figure 23 shows the inference time per sample. The time remained nearly constant throughout the test, indicating stable performance across the measurement window.

## 5. Conclusions

This study presents an approach to step detection and counting for indoor navigation using long short-term memory networks trained on smartphone sensor data. By incorporating user activity type and movement patterns into the training process, the proposed method significantly improves the accuracy of step detection across a range of real-world indoor scenarios. The generalized LSTM model achieved an average accuracy of 93%, while scenario-specific models reached up to 96%, highlighting the potential benefits of tailored models for specific motion patterns.

Our results demonstrate that neural network-based models, particularly LSTMs, can effectively capture the temporal dependencies and variability in human gait, even under complex and transitional conditions such as turning or using stairs. However, limitations remain, especially in cases involving abrupt stops or motion artifacts from non-walking activities, which occasionally lead to false positives or missed steps. Specialized models are more robust in these situations, reducing noise-induced errors and enhancing detection consistency. In order to address this, we introduced an innovative training strategy in which dedicated (activity-specific) LSTM networks were developed for distinct movement scenarios (e.g., straight walking, turning, stair transitions). Unlike traditional generalized LSTM models trained on mixed data, these specialized networks learned the subtle step-related features unique to each activity. As a result, they achieved higher overall, demonstrated improved robustness against noise, and reduced misclassifications during irregular or transitional movements. This specialization represents a key innovation of our work, highlighting how adapting LSTM training to specific activity contexts enhances detection consistency and reliability for real-world indoor navigation applications.

The methods developed in this study could find practical application in a variety of domains where precise and low-cost indoor navigation is critical. Examples include supporting couriers and delivery personnel in locating apartments within complex residential buildings, assisting patients and visitors in hospitals or large public facilities, guiding emergency responders in time-sensitive operations, and enabling context-aware services in smart buildings. In particular, the use of dedicated activity-specific LSTM networks may prove especially effective in navigation scenarios within well-mapped environments, where users encounter multiple types of movement such as walking on flat surfaces, climbing or descending stairs, and riding elevators. This represents a direct continuation of our earlier work [12], extending the approach from general activity recognition to precise step detection and step counting across diverse movement conditions.

While the current implementation provides valuable insights and strong offline performance, inference speed remains a challenge for real-time applications on mobile devices. Overall, the findings contribute to the advancement of smartphone-based indoor positioning systems and lay the groundwork for more accessible, accurate, and adaptable navigation tools in complex indoor environments. Building on this foundation, future research could explore two potential directions. First, efforts might focus on optimizing neural network architectures to enable real-time deployment on mobile devices. Approaches such as model quantization, pruning, or the adoption of lightweight alternatives (e.g., GRU or temporal convolutional networks) could be examined to reduce computational overhead and inference latency, thereby making the system more feasible for practical applications. Second, the generalization capability of the models could be strengthened by expanding the research to encompass a broader variety of users, devices, and environments. Such diversification might improve robustness to differences in data patterns, smartphone hardware, and contextual conditions, ultimately supporting more reliable and scalable indoor navigation solutions in real-world scenarios.

## Figures and Tables

**Table 1:** The table of step detection results.

Type	Real per Type	Detected per Type	Accuracy	Recall	F1-Score
Flat 10	104	95	0.93	0.85	0.89
Left Turn	107	103	0.94	0.91	0.92
Right Turn	103	100	0.96	0.93	0.95
Downstairs Transition	115	105	0.90	0.82	0.86
Upstairs Transition	116	109	0.91	0.85	0.88
Upstairs	114	104	0.92	0.84	0.88
Downstairs	102	103	0.93	0.94	0.93
Interrupted 3 × 3	105	111	0.88	0.93	0.90
Total			0.93	0.88	0.90

**Table 2:** The table of step detection results for specialized LSTMs.

Type	Real per Type	Detected per Type	Accuracy	Recall	F1-Score
Flat 10	104	104	0.99	0.99	0.99
Left Turn	107	108	0.97	0.98	0.97
Right Turn	103	100	0.97	0.94	0.95
Downstairs Transition	115	114	0.97	0.96	0.96
Upstairs Transition	116	112	0.96	0.93	0.94
Upstairs	114	113	0.97	0.96	0.96
Downstairs	102	102	0.95	0.95	0.95
Interrupted 3 × 3	105	106	0.95	0.96	0.95
Total			0.96	0.96	0.96

## Data Availability

The original data presented in the study are openly available at https://github.com/MDG369/Step-Detection-LSTM-Scripts (accessed on 29 September 2025).

## Abbreviations

The following abbreviations are used in this manuscript:

BLE	Bluetooth low energy
CEEMDAN-HT	Complete Ensemble Empirical Mode Decomposition with Adaptive Noise Hilbert Transform
CNN	Convolutional neural network
CSI	Channel state information
CPU	Central processing unit
GPS	Global positioning system
GPU	Graphics processing unit
GRU	Gated recurrent unit
IMU	Inertial measurement unit
IN	Indoor navigation
LSTM	Long short-term memory
MCCIF-SDE	Multicondition constraints and instantaneous frequency-based SD and estimation
PIR	Passive infrared
RF	Radio frequency
RSSI	Received signal strength indicator
SVM	Support vector machine
YOLO	You Only Look Once
